# Deeper genomic insights into tomato CLE genes repertoire identify new active peptides

**DOI:** 10.1186/s12864-022-08980-0

**Published:** 2022-11-17

**Authors:** Samy Carbonnel, Laurent Falquet, Ora Hazak

**Affiliations:** 1 Department of Biology, University of Fribourg, Chemin du Musée 10, 1700 Fribourg, Switzerland; 2 Swiss Institute of Bioinformatics, University of Fribourg, Chemin du Musée 10, 1700 Fribourg, Switzerland

**Keywords:** Tomato *CLE* genes, Phylogenetic analysis, Expression analysis, Root cell division arrest, SlCLAVATA2

## Abstract

**Background:**

In eukaryotes, cell-to-cell communication relies on the activity of small signaling peptides. In plant genomes, many hundreds of genes encode for such short peptide signals. However, only few of them are functionally characterized and due to the small gene size and high sequence variability, the comprehensive identification of such peptide-encoded genes is challenging. The *CLAVATA3 (CLV3)/EMBRYO SURROUNDING REGION-RELATED (CLE)* gene family encodes for short peptides that have a role in plant meristem maintenance, vascular patterning and responses to environment. The full repertoire of *CLE* genes and the role of CLE signaling in tomato (*Solanum lycopersicum*)- one of the most important crop plants- has not yet been fully studied.

**Results:**

By using a combined approach, we performed a genome-wide identification of *CLE* genes using the current tomato genome version SL 4.0. We identified 52 *SlCLE* genes, including 37 new non annotated before. By analyzing publicly available RNAseq datasets we could confirm the expression of 28 new *SlCLE* genes. We found that *SlCLEs* are often expressed in a tissue-, organ- or condition-specific manner. Our analysis shows an interesting gene diversification within the *SlCLE* family that seems to be a result of gene duplication events. Finally, we could show a biological activity of selected SlCLE peptides in the root growth arrest that was *SlCLV2*-dependent.

**Conclusions:**

Our improved combined approach revealed 37 new *SlCLE* genes. These findings are crucial for better understanding of the CLE signaling in tomato. Our phylogenetic analysis pinpoints the closest homologs of Arabidopsis *CLE* genes in tomato genome and can give a hint about the function of newly identified *SlCLEs.* The strategy described here can be used to identify more precisely additional short genes in plant genomes. Finally, our work suggests that the mechanism of root-active CLE peptide perception is conserved between Arabidopsis and tomato. In conclusion, our work paves the way to further research on the CLE-dependent circuits modulating tomato development and physiological responses.

**Supplementary Information:**

The online version contains supplementary material available at 10.1186/s12864-022-08980-0.

## Background

In plants, in addition to the classical hormones, small secreted peptides convey signals that guide cell divisions, promote specific differentiation programs and impact on hormone homeostasis and defense responses [1] (reviewed in [2]). One of the most studied groups of hormone-like peptides derived from nonfunctional precursors is the CLAVATA3/EMBRYO-SURROUNDING REGION-RELATED (CLE) family [3–6]. These short peptides control cell divisions in the shoot and root apical meristems, mediate vascular patterning during secondary growth, and are essential in root protophloem development [3, 7, 8]. In legume species, CLE peptides suppress nodulation [9]. The *CLE* genes are relatively small and encode for non-functional pre-propeptides of about 100 amino acids, containing an N-terminal signal peptide, a central variable region and a C-terminal highly conserved CLE domain. To become active peptides, additional processing, including cleavage by subtilases [10], and, often prolines hydroxylation and glycosylation are necessary [10–13]. Mature CLE peptides are secreted to the apoplast, where they are perceived by the Class XI of the leucine-rich repeats receptor-like kinases (LRR-RLKs) [14, 15]. Commercially synthetized CLE peptides can be applied exogenously to mimic the effect of overexpressed peptide genes [16]. In Arabidopsis, in addition to the CLAVATA1 receptor-like kinase, three BARELY ANY MERISTEM (BAM) receptors have been shown to perceive mature CLE peptides. These receptors have three domains: an extracellular domain, which is responsible for the binding of the ligand, a transmembrane domain, which anchors the receptor in the plasma membrane, and a cytoplasmic kinase domain, that triggers the intracellular signaling by phosphorylating downstream targets. Receptor-like kinases CLV3 INSENSITIVE KINASES (CIKs) act as co-receptors both in perceiving root-active CLE peptides and in CLV3 signaling in the shoot apical meristem [17, 18]. In addition to these cognate receptors, it has been shown in Arabidopsis, that LRR receptor-like protein (LRR-RLP) named CLAVATA2 (CLV2) creates a dimer with the pseudo-kinase CORYNE (CRN) to perceive the full range of root-active CLE peptides [15].

The genome-wide analyses of *CLE* genes have been performed in many plant genomes, including tomato, rice, wheat, maize, soybean, grape, potato and cucumber [19–22]. Due to the small gene size and high sequence variability, the annotation is challenging. Tomato (*Solanum lycopersicum*) is one of the most important crop plants that is cultivated worldwide and at the same time it is a model plant used for intensive molecular research [23]. Finding new regulators of growth and physiological adaptations is crucial for improving tomato plants to achieve better yields and increased tolerance to environmental stresses.

It has been previously reported, that in the tomato genome there are 15 *SlCLE* genes [22] which is relatively little compared to other plant species. The objective of this study was to perform a deeper analysis of CLE family in tomato which would be essential to obtain a complete overview of these molecular players to allow to dissect later their roles in growth and physiological responses. We used an improved approach to gain deeper genomic insights into the CLE repertoire in this fleshy fruit crop plant. We identified 37 new *SlCLE* genes expressed in different tissues of tomato plants. The biological activity of the selected peptides tested by root growth assays showed functional conservation with the orthologs from Arabidopsis. Finally, we found that the perception of *SlCLEs* in the roots depends on the receptor-like protein *SlCLAVATA2,* demonstrating that also the mechanism of sensing of these peptides is highly conserved.

## Results

### Genome-wide identification of 37 new *SlCLE* genes

**Fig. 1:**
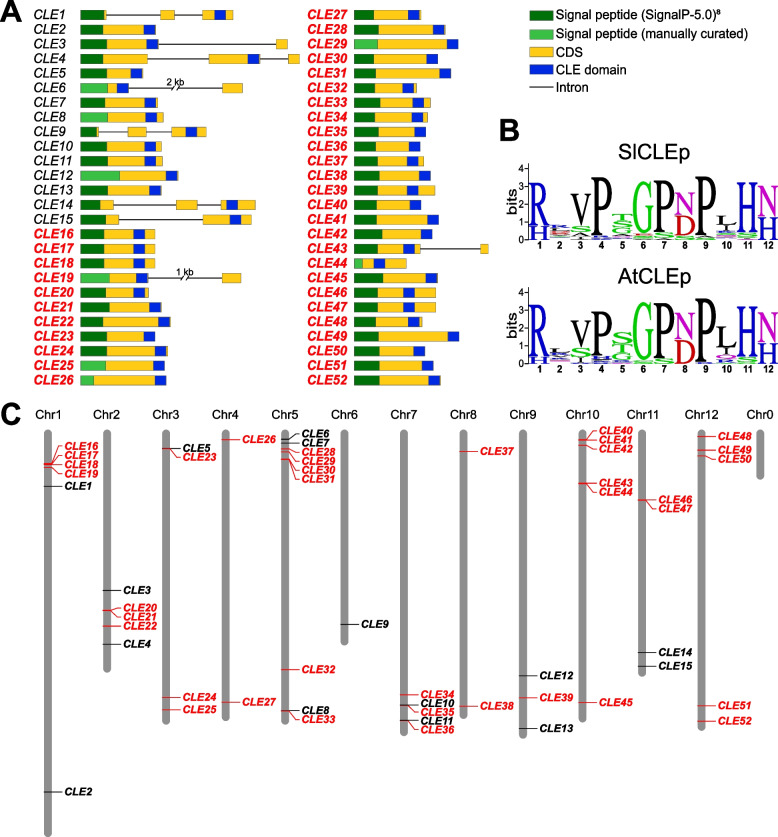
*CLE* genes identified in the tomato genome. **A**. The gene structure of the tomato *CLE* genes. The names in red indicate the new *CLE* genes uncovered in this study **B**. Sequence logo of the conserved CLE domain in tomato and Arabidopsis using WebLogo (https://weblogo.berkeley.edu/logo.cgi). The height of the bars represents the conservation value of each amino acid at the given position. **C**. Chromosomal location of the tomato *CLE* genes

The previous genome-wide analysis revealed only fifteen *SlCLE* genes [22] and further attempts failed to uncover additional genes [20, 21]. In our study, we applied a combined bioinformatic approach to search for the additional *SlCLE* genes using the most recent versions of the tomato reference genome SL3.0 and SL4.0 [24]. Firstly, we performed an iterative tBLASTn search on the full tomato genome, using known Arabidopsis *CLE* genes and searched sequences from closely related Solanaceae species. This analysis revealed forty *CLE* genes, including twenty-five new *SlCLE*s. Secondly, we used a Hidden-Markov-Model, that resulted in fifty-two *CLE* genes, including all found by tBLASTn and twelve additional new *SlCLE*s. The initially larger number of *SlCLE* candidate genes was manually analyzed for the presence of all landmarks of *CLE* gene (Fig. 1A).

We mapped the *SlCLE* genes on tomato’s chromosomes (Table 1, Fig. 1C). We numbered the identified *SlCLE* genes as follows: the previously reported fifteen genes are numbered *SlCLE1-SlCLE15*. The newly identified genes (*SlCLE16* to *SlCLE52*) are numbered according to their chromosomal location, starting from chromosome 1 (Table 1). *SlCLE* are diversely present on all 12 chromosomes in tomato, from a single gene on chromosome 6 to up to nine genes on chromosome 5 (Fig. 1C). The fact that several *SlCLE* genes are located in high proximity with each other’s, forming gene clusters, and showing high sequence similarity, suggests that they arise from tandem gene duplication events [25].

**Table 1:** Chromosome locations and peptide sequences of *SlCLEs* uncovered in this study

**Name**	**Predicted CLEp**	**Chromosome**	**Start**	**End**
**CLE1**	RQIPTGPDPLHH(N)	1	11619943	11620437
**CLE2**	REVPSSPDPLHN	1	81529230	81529475
**CLE3**	RRVPSEPDPIHN	2	35723792	35724463
**CLE4**	RRVPTGPNAIHN	2	47936570	47937280
**CLE5**	HLVPGGPNPLHN	3	3374293	3374493
**CLE6**	RRVPNGPDPIHN	5	1311175	1313680
**CLE7**	RKVRKGSDPIHN	5	2204552	2204803
**CLE8**	HDVPSGANPVQN	5	63087495	63087764
**CLE9**	REAPMSPDPLHH(H)	6	43447059	43447467
**CLE10**	RVVPGGPDSQHH	7	61751575	61751838
**CLE11**	RVAPQGPDAQHH	7	65194792	65195058
**CLE12**	HEVPSGPNPISN	9	55141751	55142068
**CLE13**	HEVPSGANPESN	9	67120505	67120768
**CLE14**	HKPPSGPNPNGN(H)	11	49841730	49842297
**CLE15**	RGVPAGPDPLHH(N)	11	52945095	52945649
**CLE16**	HDVPAGPSPSHN	1	6946626	6946868
**CLE17**	HDVPAGPSPSHN	1	7005125	7005367
**CLE18**	HDVPAGPSPSHN	1	7027437	7027679
**CLE19**	RRVPNGPDPIHN	1	7704510	7706013
**CLE20**	RVSPGGPDPHHH	2	40195502	40195723
**CLE21**	RVAPGGPDPQHH(N)	2	40204594	40204857
**CLE22**	HEVPSGPNPISN	2	43800735	43801028
**CLE23**	RLVPSGPNPLHN	3	3376485	3376727
**CLE24**	RRVKRGSDPIHN	3	59983924	59984208
**CLE25**	RLVPTGPNPLHH	3	62820859	62821140
**CLE26**	RLVHTGPNPLHN	4	1449560	1449838
**CLE27**	RRVPNESDPLHN	4	61069201	61069419
**CLE28**	RRVPSCPDPLHN	5	3515405	3515701
**CLE29**	RLVPTGPNPLHH	5	4183891	4184229
**CLE30**	RSIPSGPNPLHN	5	5853919	5854191
**CLE31**	RLVPSGPNPLHN	5	5856133	5856446
**CLE32**	RRVPTGSNPLHN	5	53722541	53722744
**CLE33**	HDVPSGPNSPIH	5	63089819	63090067
**CLE34**	RRVPTGPNPLHN	7	59399859	59400098
**CLE35**	RRSPGGPDPKHH	7	61762724	61762957
**CLE36**	RLSPGGPDPKHH	7	65205136	65205351
**CLE37**	REVPTGPDPLHH(H)	8	4071288	4071515
**CLE38**	RIVPGGPNPLHN	8	62042236	62042481
**CLE39**	HDVPTGPSPSHN	9	60775375	60775638
**CLE40**	RLSPGGPDPRHH	10	1482971	1483189
**CLE41**	RLSPRGPNPKHH	10	1486893	1487168
**CLE42**	RVAPGGPDPKHH	10	2690033	2690287
**CLE43**	RTAPTGPSPIHH	10	11338810	11339245
**CLE44**	RTVPAVPNPIHH	10	11387275	11387445
**CLE45**	RKVRTGPNPLHN	10	61194096	61194368
**CLE46**	RTVPTGPNPIHH	11	15143289	15143555
**CLE47**	RTVPTGPNPIHH	11	15193925	15194191
**CLE48**	RRIPTGSNPLHN	12	744075	744296
**CLE49**	RISPGGPDPKHH	12	3799102	3799443
**CLE50**	RLSPGGPDPRHH	12	5056108	5056338
**CLE51**	HAVPGGPNPLHN	12	61900450	61900707
**CLE52**	HSVPSGPNPESN	12	65362393	65362674

To investigate the gene structure of tomato *CLE*s, the exon-intron composition was predicted based on sequence homologies (Fig. 1A). In addition, we used publicly available RNAseq datasets [26–30], from root, shoot and fruit samples, to support these gene structure predictions. Reads were mapped on the anticipated coding region of 28 *CLE* genes out of the 37 newly uncovered loci (Fig. 1A). Overall, the tomato *SlCLE*s have a single CLE domain in the 3′ of the coding region and rarely include any intron (Fig. 1A). In the case of *SlCLE31*, an insertion of a single nucleotide in the tomato genome SL4.0, which is not present in the version SL3.0, creates a frameshift in the CDS suggesting that it is a pseudogene. However, Sanger sequencing of this particular locus confirmed the correctness of the sequence in the SL3.0 genome.

Furthermore, to evaluate to what extend the CLE motif is conserved between Arabidopsis and tomato, we created sequence logos (Fig. 1B). We found that the CLE domain is extremely well conserved, including the prolines at positions 4, 6, and 9, as well as the arginine at position 1, glycine at positions 6, and histidine-asparagine/histidine at positions 11–12.

Tomato (*Solanum lycopersicum*) and potato (*Solanum tuberosum*) belong to the same genera and share high gene sequence similarities [23]. A recent study reported about 41 *CLE* genes in potato [19]. We used the sequences of potato *CLE* genes described in this work to perform a phylogenetic analysis with tomato *CLE* genes (Supplemental Fig. 1). Except for *StCLE2* and *StCLE5*, we found orthologous for all the other CLE genes in the potato genome, which indicates that both studies identified most of the *CLE* genes.

### Phylogenetic analysis of CLE receptor genes

The mature CLE peptides act as ligands to a specific group of LRR-RLKs. To obtain a better overview on CLE signaling components in tomato, we analyzed genes encoding for CLE receptors in several eudicot species, including Arabidopsis and tomato. To this end, we performed a search for the homologs of Arabidopsis *CLV1*, *BAM1*, *BAM2*, *BAM3,* and *PXY*. As previously reported in tomato [31], we found one copy of *CLV1*, four *BAM* homologs, two *PXY*-like genes, one *PXL1,* and one *PXL2* (Supplemental Fig. 2A). In contrast to the *CLE* genes, the number of CLV1-type receptors is similar between Arabidopsis and tomato. One special case, *BAM4* is present in the tested eudicots except in Arabidopsis. Considering that separation of the *Fabaceae* (Medicago and Lupinus) and the *Brassicaceae* (Arabidopsis) is more recent than with the *Solanaceae* (Tomato, Potato), *BAM4* was likely lost in *Arabidopsis thaliana* during evolution. Further, we looked at the conservation of the receptors at the protein sequence level (Supplemental Fig. 2B). Overall, the tomato receptors show a high sequence similarity to their Arabidopsis orthologs (71,4% for BAM1/2, 62,8% for BAM3, 61,9% for CLV1), notably in the kinase domain. Curiously, 25 amino-acids are deleted in the extracellular domain of the BAM3s from the Solanaceae, which corresponds to one missing leucine-rich repeat. According to a recent publication [32], this leucine rich repeat is situated just above the binding site of AtCLE9/10p to AtBAM1, and could potentially play a role in ligand binding selectivity.

### Expression analysis of *SlCLE*s

**Fig. 2:**
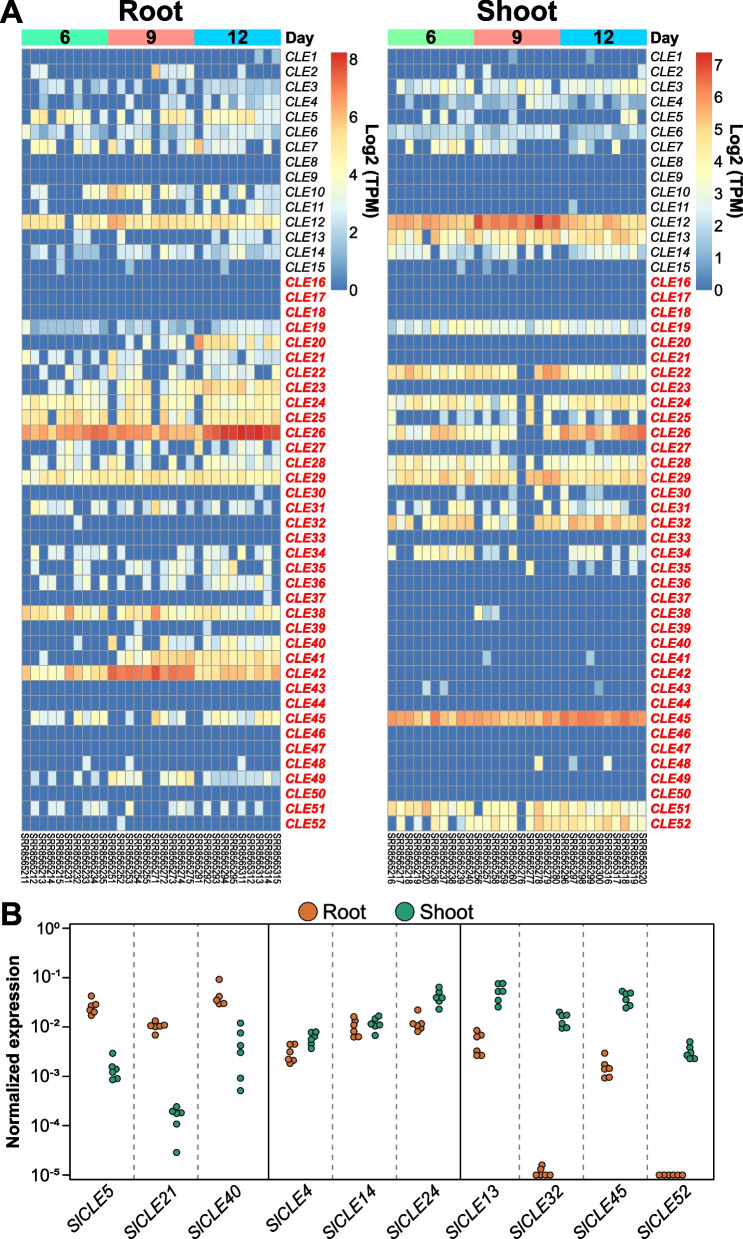
Expression analysis of *SlCLE* genes in root and shoot tissues [28]. **A** Heatmaps of log (TPM) of tomato *CLE* genes in the root (left) and the shoot (right) at 6, 9, and 12 days after plantation from tomato grown in pots. **B** Expression of selected *SlCLE*s in root and shoot tissues by qPCR from 3 weeks old tomato plants grown in hydroponic conditions

In order to confirm that the newly identified genes are truly expressed in tomato, we performed an analysis of publicly available RNAseq datasets from root and shoot samples, from drought stress-exposed plants and from fruits at different stages of development [26–30]. Remarkably, based on this analysis, it appears that the majority of *SlCLEs* shows predominant expression in root tissues, while some are shoot-specific or evenly expressed in both (Fig. 2A). Using qPCR, we could confirm that *SlCLE5*, *SlCLE21*, *SlCLE40* show higher expression in the tomato root tissues, while *SlCLE13*, *SlCLE32*, *SlCLE45,* and *SlCLE52* are more expressed in the shoot tissues (Fig. 2B).

Later, we looked into the fruit transcriptome to analyze whether *SlCLE* genes are expressed during fruit development [26]. In this study, wild type M82 and yellow-fruited *yft1* mutant fruits were sampled at different developmental time points, from 35 to 60 days-post-antherisation. We found that *SlCLE12*, *SlCLE30*, *SlCLE31*, *SlCLE34*, and *SlCLE38* are the most expressed in tomato fruits independently of the genotype, whereas *SlCLE5, SlCLE11, SLCLE51* induction is impaired in the yellow-fruited *yft1* mutant (Supplemental Fig. 3A). These results suggest, that *SlCLE* genes could play a key role during tomato fruit ripening.

Numerous studies showed that in Arabidopsis the CLE peptides mediate abiotic stress signals (summarized in [33]), for example, CLE25 peptide in Arabidopsis was shown to be induced during dehydration, moving from root to shoot as a mobile signal, triggering ABA biosynthesis and stomatal closure [34]. Therefore, we wanted to test, whether some *SlCLE* genes are up-regulated under drought stress conditions. First, we analyzed a previously published RNA-seq dataset [27]. In this study, the drought-sensitive (M82) and drought-resistant (IL9–1) tomato seedlings at the five-leaf stage were challenged with prolonged drought during 10 days to identify miRNAs and mRNAs that respond to this stress. In our analysis, we could find several genes that are specifically expressed under drought in tomato leaves (Supplemental Fig. 3B) [27]. *SlCLE1*, *SlCLE12*, *SlCLE32, SlCLE45* and *SlCLE52* showed an increased expression (Supplemental Fig. 3B), suggesting that they could be involved in adaptive responses to water deficit. Next, we tested whether these genes can be quickly up-regulated under short osmotic stress. To this end, hydroponically grown tomato plants were treated with a 15% PEG6000 solution for 1 h, and roots and shoots samples were collected separately. Since Dehydrins (DHN) play a key role in plant response and adaptation to water deficit conditions and are accumulated during drought stress, we used the *SlDehydrin (SlDHN)* (*Solyc02g084850*) expression as a control to monitor the effect of water deficit in our experiment. After 1 h, *SlDHN* was strongly upregulated both in root and shoot tissues of treated tomato plants (Supplemental Fig. 3C). However, we could not detect a significant induction for those *SlCLE* genes (Supplemental Fig. 3C). Further, we questioned whether similarly to Arabidopsis, the tomato orthologs of *AtCLE25* are upregulated in roots to mediate a dehydration response like it has been demonstrated in Takahashi et al. 2018 [34]. We could not detect any significant induction in *AtCLE25* orthologs in tomato under this short osmotic stress (Supplemental Fig. 3D). One possibility is that our experimental settings did not trigger similar osmotic stress like reported in [34] and [27]. Another possibility is that in tomato, none of the *AtCLE25* orthologs are involved in mediating drought responses or this regulation is without their transcriptional activation.

### Diversification of *SlCLE*s

**Fig. 3:**
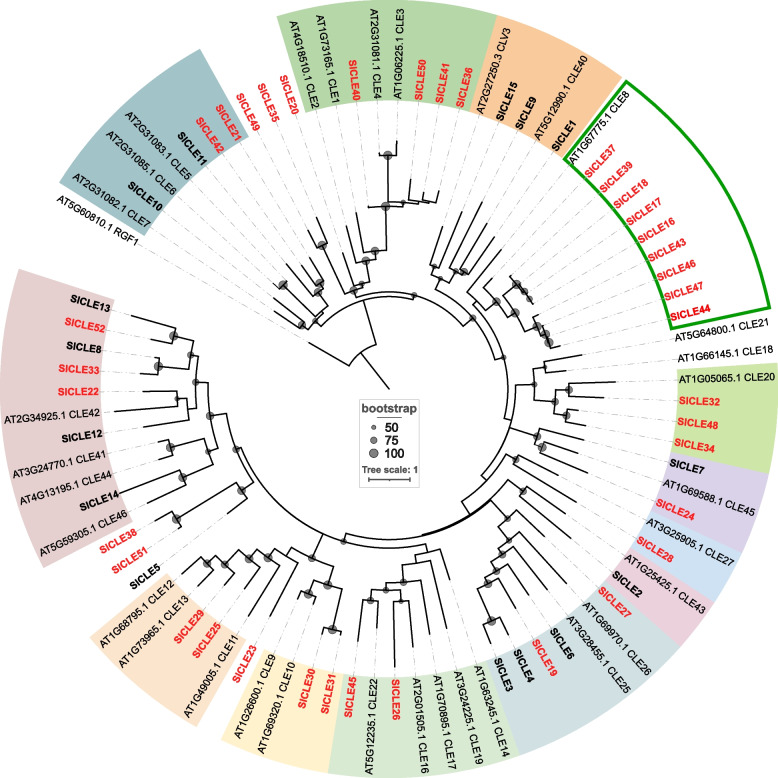
Phylogenetic tree of the full-length CLE proteins from tomato and Arabidopsis. The groups of proteins sharing high similarity (clusters) are highlighted by background colors. The names in red indicate the new *CLE* genes uncovered in this study. Nodes supported by bootstrap values superior to 50 are indicated by dots of size proportional to the bootstrap values

To explore the diversification of the tomato *CLE* genes, we created a phylogenetic tree of the full-length proteins from tomato and Arabidopsis (Fig. 3). This analysis revealed gene sub-groups that are conserved in both plant species and define orthology, as well as showed unique genes which could pinpoint *CLE* diversifications in tomato or losses in Arabidopsis. Interestingly, we found nine homologs of Arabidopsis *CLE8* in tomato (Fig. 3), whereas only two were discovered in potato (supplemental Fig. 1) suggesting a very recent surge in their duplications. These nines genes are present on five different chromosomes (Fig. 1). We can speculate, based on the chromosomal location (Fig. 1D) and sequence similarity (Fig. 3), that these genes probably arise from a mix of tandem duplications (for *SlCLE16/17/18*) and disperse duplication (*SlCLE43/44* with *SlCLE46/47*).

In Arabidopsis, CLE8 peptide is expressed and acts specifically during embryo and endosperm development [35], but the roles of the nine orthologs in tomato are yet to be uncovered. For phloem-associated Arabidopsis CLE peptides (*AtCLE25*, *AtCLE26*, *AtCLE45*) we found seven orthologs in tomato, which also suggests the diversification of the phloem genes.

### A conserved effect of *SlCLE* peptides on root apical meristem

**Fig. 4:**
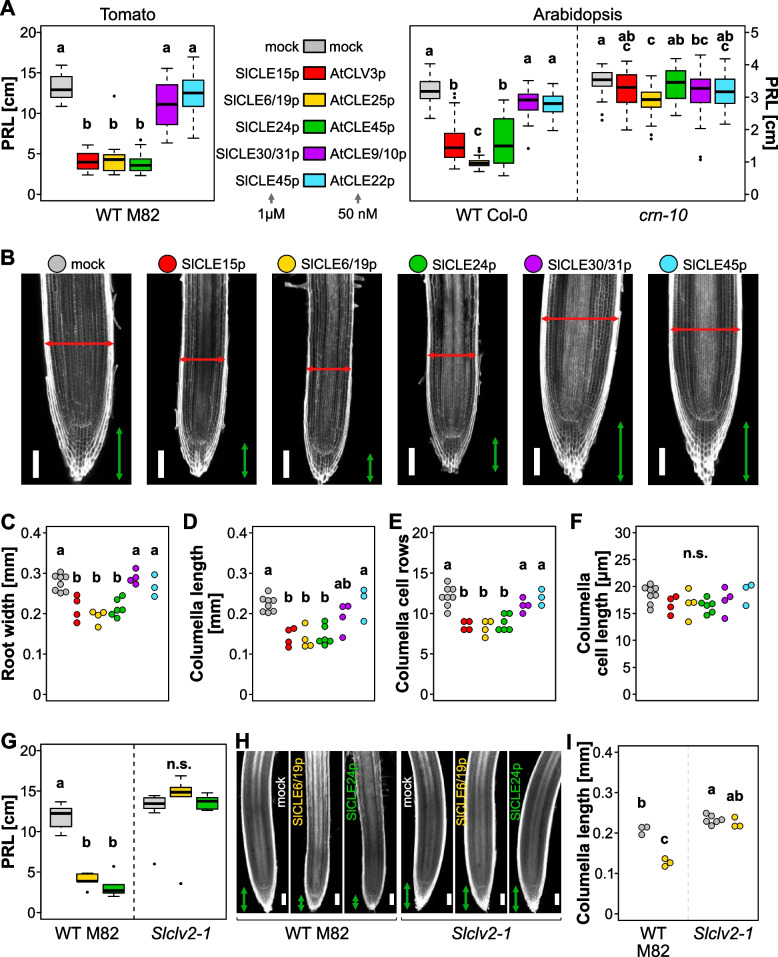
Functional conservation of root active CLE peptides in tomato and Arabidopsis. **A**. Effect of Arabidopsis and tomato orthologous CLE peptides on the primary root length (PRL). **B**. Representative confocal images of tomato primary root tips grown on mock or indicated SlCLE peptide containing medium. The cell walls are stained with calcofluor white. Red and green arrows indicate root width columella length, respectively **C-F**. Quantification of the indicated root tip morphology characteristics from images show in B. Letters indicate different statistical groups (ANOVA, followed by post-hoc Tukey test). **G.** Primary root length of wild type and *Slclv2* mutant grown in presence of SlCLE6/19p and SlCLE24p. **H**. Representative confocal images of wild type and *Slclv2* primary root tips grown on mock or indicated SlCLE peptide containing medium. The scale bars in **B** and **H** correspond to 100 μm

After confirming the expression for the majority of predicted CLE peptides coding genes, we wanted to check their activity *in planta*. It has been shown, that in Arabidopsis 20 out of 32 peptides affect the primary root growth, leading to root meristem arrest [15, 36]. To study the activity of orthologous CLE peptides in tomato, we tested their capacity to inhibit root growth. For this purpose, we selected CLE peptides from different well-supported orthologous subgroups in tomato and Arabidopsis. For example, the treatment with AtCLV3, AtCLE25, and AtCLE45 peptides at 50 nM triggers a strong reduction of the primary root length (Fig. 4A right side), and this response depends on the pseudo-kinase CORYNE and the receptor-like protein CLAVATA2 [15]. However, the AtCLE9/10 and AtCLE22 peptides cause a much smaller root growth inhibition effect in the wild-type. Because tomato roots are much thicker than Arabidopsis roots and have an additional apoplastic barrier in exodermis, the tomato root meristem is less sensitive to external application of CLE peptides. Therefore, we applied SlCLE peptides at a concentration of 1 micromolar. We could observe, that in tomato roots, SlCLE15, SlCLE6/19 and SlCLE24 peptides (orthologous of AtCLV3, AtCLE25, and AtCLE45, respectively) led to a strong reduction of the primary root growth (Fig. 4A left side). In contrast, SlCLE30/31 and SlCLE45 peptides (orthologous of AtCLE9/10 and AtCLE22, respectively) treatment did not trigger a significant reduction of the primary root length. This result indicates that the amino-acid composition of CLE peptides is important for their biological activity in the root; and that there is a conservation of the biological activity of these CLE peptides between Arabidopsis and tomato, two evolutionary separated species.

To have more insight into the effect of SlCLEs on tomato roots, we analyzed the morphology of the root tips (Fig. 4B). We observed in three treatments (SlCLE15, SlCLE19, SlCLE24) not only the decreased root length but also the reduction in the root diameter and the columella length. We also looked at the number of columella layers and the columella cell length to understand whether the treatment affects cell division or cell elongation. Root-active SlCLE peptides treatment led to a reduction of columella layers, but not their average cell length, suggesting that cell division is primarily affected (Fig. 4C-F).

Next, we asked whether this inhibitory effect on the root is mediated by orthologous receptor-like kinases? To answer this question, we tested the loss-of-function mutants *clv1 bam1 bam4* and *clv2* [31] for their root sensitivity to SlCLE peptides. The mutant *clv1 bam1 bam4* showed a strong sensitivity to SlCLE24 peptide (Supplemental Fig. 4). However, *clv2* roots were absolutely blind to the high concentrations of the peptide in the media, strongly suggesting that this response is *SlCLV2*-dependent (Fig. 4 G-I). This result reinforces the claim, that CLE peptides have a conserved root activity across plant species and that the perception mechanism is similar.

## Discussion

Signaling mediated by CLE peptides evolved gradually in all land plant lineages [37]. The precise control of the shoot apical meristem stem cell niche by CLV3-CLV1 module is the most ancient pathway, whereas additional CLE genes and receptor complex components evolved later, with establishing vascular plants [37]. It seems, that the possible ancestral function of CLV3-like peptides was to suppress the proliferation of the shoot apical meristem in early land plants (bryophytes).

Our study aimed to re-analyze the repertoire of tomato *CLE* genes in order to build a better basis for the future functional studies. We showed that some *SlCLE*s are root-specific, while others are highly induced during fruit development or following prolonged drought stress. One of the limitations of our study was the number of RNAseq datasets that we analyzed and that does not include neither all tissues and developmental stages of tomato nor pathogen infection or stresses beyond prolonged drought. We therefore could not obtain full evidence for the expression of all new *SlCLE* genes.

Among the previously described *SlCLE*s, *SlCLV3* and *SlCLE9* encode for peptides that control the stem cell proliferation and shoot apical meristem size. Remarkably, the tomato domestication mutation *fasciated (fas)* that led to the increased fruit size, is a result of disruption of the *SlCLV3* promoter that led to the reduction in the gene [7]. The *SlCLE9* is the closest paralog of *SlCLV3* and can actively compensate for the absence of *SlCLV3* to buffer the impact on the stem cell niche [7, 31]. The unraveling of additional tomato *CLE* genes and more careful phylogenetic analysis is necessary to fully understand the role of these conserved ligands in tomato development and adaptation to the changing environment. Our analysis did not find any additional homologs of *SlCLV3*, but for all other previously described *SlCLE*s we found additional genes that might have redundant function. For example, in our analysis *AtCLE42* that was previously reported as having three closest homologs in tomato (*SlCLE8*, *SlCLE12* and *SlCLE13*) [22], in fact has three additional genes encoding for homologous peptides: *SlCLE22*, *SlCLE33* and *SlCLE52* (Fig. 3) which indicates a diversification of this subgroup of *SlCLE*s in tomato genome. We found additional evidences of gene diversification events among *SlCLE* genes and further research will shed light on the biological meaning of them.

In our work, to study the activity of tomato CLE peptides, we used unmodified synthetic peptides. It has been shown, that SlCLV3 and SlCLE9 undergo arabynosylation. While glycosylated, these peptides are active at 60 nM concentration [7]. Moreover, it has been demonstrated that the biological activity of Arabidopsis CLV3 gradually increases in mono-, di- and triarabinosylated CLV3 glycopeptides, becoming equally active with non-modified peptide at 1 μM concentration [38]. The synthesizing of the complex arabinose chain is technically difficult and only a few laboratories in the world established such synthesis [38], therefore in our study we decided to use the unmodified peptides at 1 μM concentration. It is plausible, that the effect of glycosylated SlCLEs on the root meristem will be visible at a much lower concentration.

We observed that the activity of SlCLE peptides in repressing root growth is conserved and relies on the cell divisions arrest. This effect induced by root-active SlCLEs requires the ortholog of receptor-like protein RLP10 also called CLAVATA2, indicating the conservation of CLE sensing mechanism between tomato and Arabidopsis.

It has been recently shown, that the N-terminal part of the CLE domain, containing RLV residues, is essential for peptide recognition by the Arabidopsis receptor BAM1 [32]. In tomato SlCLEs this domain is highly conserved, which suggests that the mechanisms of sensing of tomato CLEs can be similar to one in Arabidopsis.

In general, genes encoding for small secreted peptides are often overlooked and omitted in the genome annotations because their conserved motifs are short. The approach usually used for the identification of such genes is BLAST [39]. However, when the CLE prepropeptide is used as a query, the signal peptide and the variable domain with low sequence conservation prevent obtaining a good BLAST result. A recent study that aimed to identify *CLE* genes in 69 plant species with a newly developed machine-learning-aided method [21], did not uncover additional tomato *CLE*s. The hidden Markov models (HMMs) [40] was shown to be very efficient to scan plant genomes for new genes encoding for small secreted peptides. In our case, this approach combined with the multistep procedure for validation, was successful in the identification of 37 new *SlCLE*s. Our work lies a foundation for the future functional analysis of these genes.

## Conclusions

Our study showed that tomato genome encodes for a larger number of *SlCLE*s than thought before. In addition, our analysis revealed that the receptor-like kinase BAM4 was lost in the Arabidopsis genome during evolution and the function of this gene in tomato remains to be uncovered.

The phylogenetic analysis and the clustering of the *SlCLE* genes with the Arabidopsis orthologs allowed us to detect multiple diversification events. For example, we found nine orthologs of *AtCLE8* [35] in tomato genome and the evolutionary meaning of this event has to be investigated further. In conclusion, our work draws a more precise picture of the components of CLE signaling in this fleshy fruit crop plant paving a path for new discoveries.

## Methods

### *SlCLE*s identification

#### Iterative tBLASTn

All previously described *Arabidopsis thaliana* CLE full-length protein (pre-propeptide) sequences were retrieved from TAIR and used as queries to search by tBLASTn in *Solanum lycopersicum* genome SL3.0 and SL4.0 in the plant section of the EnsemblGenome and Solgenomics network databases [41–43]. The hits were then used to search by BLASTp in closely related species of the Solanaceae family (*Nicotiana attenuata* NIATTr2, *Solanum tuberosum* SolTub_3.0*,* and *Capsicum annuum* ASM51225v2). The newly identified CLE proteins were exploited to identify by tBLASTn additional similar sequences in tomato’s genome, which were then used to search again in the above Solanaceae-species genomes. Between each iteration, candidate loci were individually confirmed based on the CLE domain sequence and the presence of a signal peptide sequence in 5′.

#### Hidden-Markov-model approach

A list of 256 CLE proteins obtained in multiple species (*A. thaliana, N. attenuata*, *S. tuberosum*, and tomato sequences found in 1.1; *Medicago truncatula* sequences were retrieved from MtSSPdb [44], *Populus trichocarpa* and *Brachypodium distachyon* sequences were obtained by BLASTp in EnsemblPlants with AtCLEs as query) was aligned with MEGA X [45] and used to build an HMM with HMMER3 [46]. The HMM was used to search *S. lycopersicum* SL4.0 genome with Genewise [47] (the genome was split in chunks of 9 million bp with EMBOSS splitter & seqretsplit [48]). This led to a list of 61 CLE candidates that was concatenated with the 40 CLE of found in 1.1. After manual cleaning and removing duplicates, we confirmed a clean list of 57 *CLE* candidates.

#### Candidate verification

The gene structure of the 57 candidate *CLE* was verified by tBLASTn and BLAT [49] against the SL3.0 genome as in 2.1.1 and by manual evaluation of the resulting hits for the correctness of their exon-intron structure. Five pseudogenes could be identified (with in-frame stop codons or no initiator methionine), leaving a final list of 52 *CLE* genes.

### Transcriptomic analysis

We selected four publicly available RNAseq and TRAPseq datasets to search for expression clues of the CLE genes in various tissue types of *S.lycopersicum* M82: RNAseqA [27], RNAseqD [26], RNAseqF1 and RNAseqF2 [28], TRAPseq [50].

The selected samples of all the four datasets were remapped to the SL4.0 genome assembly with bwa [51] and samtools [52] to obtain sorted bam files. A Bed file containing the CLE gene positions was created (CLEgene.bed) and used to count the reads per gene with bedtools multicov [53]. A heatmap of the logTPM (transcripts per million) for CLE genes counts over all genes was created with a custom-made R script (script) for each dataset.

### Phylogenetic analysis

Alignments of the CLE proteins found in 1.1 and the extracellular domain of receptors retrieved by BLASTp in the TAIR and EnsemblPlants databases were performed in MEGA X [45], using ClustalW (Fig. 2) or MUSCLE (Fig. S1-S2), and manually corrected. The phylogenetic trees were generated by IQTREE with 1000 bootstrap replicates [54], and visualized with iTOL [55]. Multi-sequence alignment profile was visualized with alignmentviewer.org. All the sequences can be found in Dryad repository.

### Plant material and treatments

#### Mutants and seed sterilization

Seeds of *Solanum lycopersicum* M82 were surface-sterilized with a sterilization solution (2.5% sodium hypochlorite, 0.1% Tween-20) for 20 minutes. Seeds of *Arabidopsis thaliana* Col-0 were surface-sterilized with 70% ethanol and 0.05% Triton-X100 solution for 3 minutes. Immediately after, the seeds were washed with sterile distilled water five times. Tomato (*Slclv1-*a2, *Slbam1-*a1, *Slbam4-*a2 and *Slclv2–5*) and Arabidopsis (*Atcrn-10*) mutants are CRISPR-mediated mutants previously described [8, 31].

#### Root assays

*S. lycopersicum* sterilized seeds were placed on 24 cm square plates containing 1 μM of the indicated SlCLE peptide. After 2 days in the dark, plates were placed vertically in 16 h light / 26 °C – 8 h dark / 24 °C cycles for a week. *A. thaliana* sterilized seeds were grown onto 12 cm square plates containing 50 nM of indicated AtCLE peptides. After 2 days in the dark at 4 °C, plates were placed vertically in 16 h light– 8 h dark cycles at 22 °C for a week. The plates were scanned at high resolution, and primary root length was measured with the “simple neurite tracer” tool on Fiji (www.imagej.net). All CLE peptides are synthetic un-modified peptides at > 75% purity (www.genscript.com) solubilized in water at 10 mM stock concentration.

#### Tomato drought stress assay

The assay was modified from a published protocol of hydroponically grown tomato [56]. In brief, sterilized tomato seeds were placed on moistened blotting paper and kept in dark at 26 °C for 3 days. Germinated seeds were placed in Eppendorf-type tubes with cut-end filled with 0.6% water-agar in 16 h light / 26 °C – 8 h dark / 24 °C cycles and high humidity environment for 1 week. Then, the seedlings were transferred to hydroponics containers, in which the roots grow in an oxygenated Hoagland solution in darkness. The nutritive solution was renewed every week. After 3 weeks, 1 day after replacing the nutrient solution, drought stress was induced with a fresh solution supplemented with 15% PEG-6000. Three different containers were used for the experiments generating each 2 biological replicates. Each biological replicate is a pool of 2 to 3 plants from the same container. The root samples contain all the root system coming out of the Eppendorf. The shoot samples contain all the leaves and around 5 cm of stem harboring the shoot apical meristem, thus these samples do not contain the main stem which has been strongly lignified.

Handling of transgenic plants was performed in accordance with the guidelines and regulations of the Department of Biology University of Fribourg. All the transgenic plants were carefully collected after experiment and treated as biohazard.

### Quantitative RT-PCR of tomato CLE genes

Plant tissues were rapidly shock frozen in liquid nitrogen. Frozen samples were grinded using mortar and pestle. Total RNA was extracted using the Spectrum Plant Total RNA kit (Sigma). The remaining DNA was eliminated by DNAse I treatment (Jena-Bioscience) and with a 2 M LiCl precipitation. The absence of the genomic DNA in the RNA samples was tested by PCR. cDNA synthesis was performed using the SensiFAST cDNA synthesis kit (meridian). Quantitative PCRs were performed using Fast Start Universal SYBR-green Master (Roche), with primers indicated in Supplemental Table 1. The thermal cycler (Mic qPCR Cycler, biomolecular systems) conditions were: 95 °C 2 min, 45 cycles of 95 °C 15 s, 58 °C 10s, 60 °C 50s, followed by a dissociation curve analysis. The expression level was normalized to Actin on 6 biological replicates.

### Microscopy

About 1 cm of the primary root tips of one-week-old tomato seedlings were fixed with 4% paraformaldehyde in a 1xPBS solution for a minimum of 6 hours. After 2 washes in 1xPBS, the samples were cleared in a ClearSee solution [57] for 1 week. Subsequently, to visualize the cell walls, the calcofluor white staining was performed with 0,02% calcofluor-white dissolved in the ClearSee solution for 2 days, followed by two washing steps with ClearSee. Samples were incubated in ClearSee solution for a minimum of 2 weeks before imaging. Images were taken with a confocal laser scanning microscope (Leica SP5). The calcofluor-white stained cell walls were excited at 405 nm and emitted light detected at 415-500 nm. These images were used to quantify root width in the differentiation zone, columella length and cell number in Fiji (www.imagej.net).

### Statistics

Statistical analysis was performed using R (version 4.0.2) after log transformation of the data. Statistical significance was analyzed by ANOVA, and followed by a post-hoc Tukey test to determine the different statistical groups.

The list of the software and main parameters are listed in Supplemental Table 2.

## Supplementary Information

**Additional file 1.**

**Additional file 2.**

## Data Availability

The datasets and sequences supporting the conclusions of this study are available in the Dryad repository https://datadryad.org/stash/share/aAsAK9iTNaSzUfpzICKCiLBiVVJmCWOlqeWAhFypTgg. The plant materials are available upon request from the corresponding author.
