# Supplementary material for: Deeper genomic insights into tomato CLE genes repertoire identify new active peptides
Source: BMC Genomics. 2022 Nov 17;23:756. doi: 10.1186/s12864-022-08980-0 (PMC9670457; doi:10.1186/s12864-022-08980-0)
Supplement: Supplementary file 1 — Additional file 1. [file 12864_2022_8980_MOESM1_ESM.pdf]

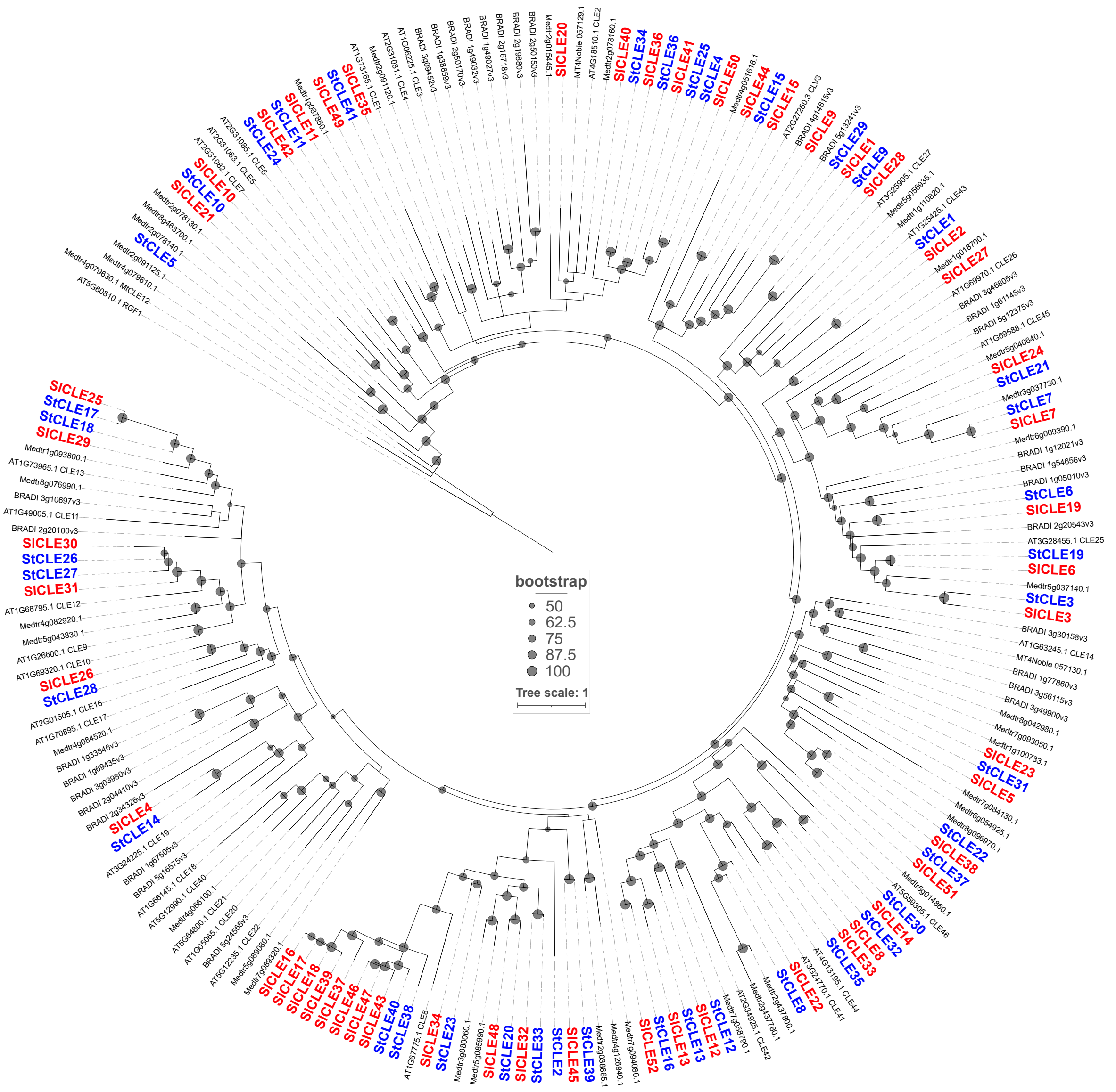

Supplemental Figure 1. Sequence homology between tomato and potato CLE proteins. Phylogenetic tree of full-length CLE proteins from tomato (red), potato (blue), *Arabidopsis thaliana*, *Medicago truncatula*, *Brachypodium distachyon*. Nodes supported by bootstrap values superior to 50 are indicated by dots of size proportional to the bootstrap values.

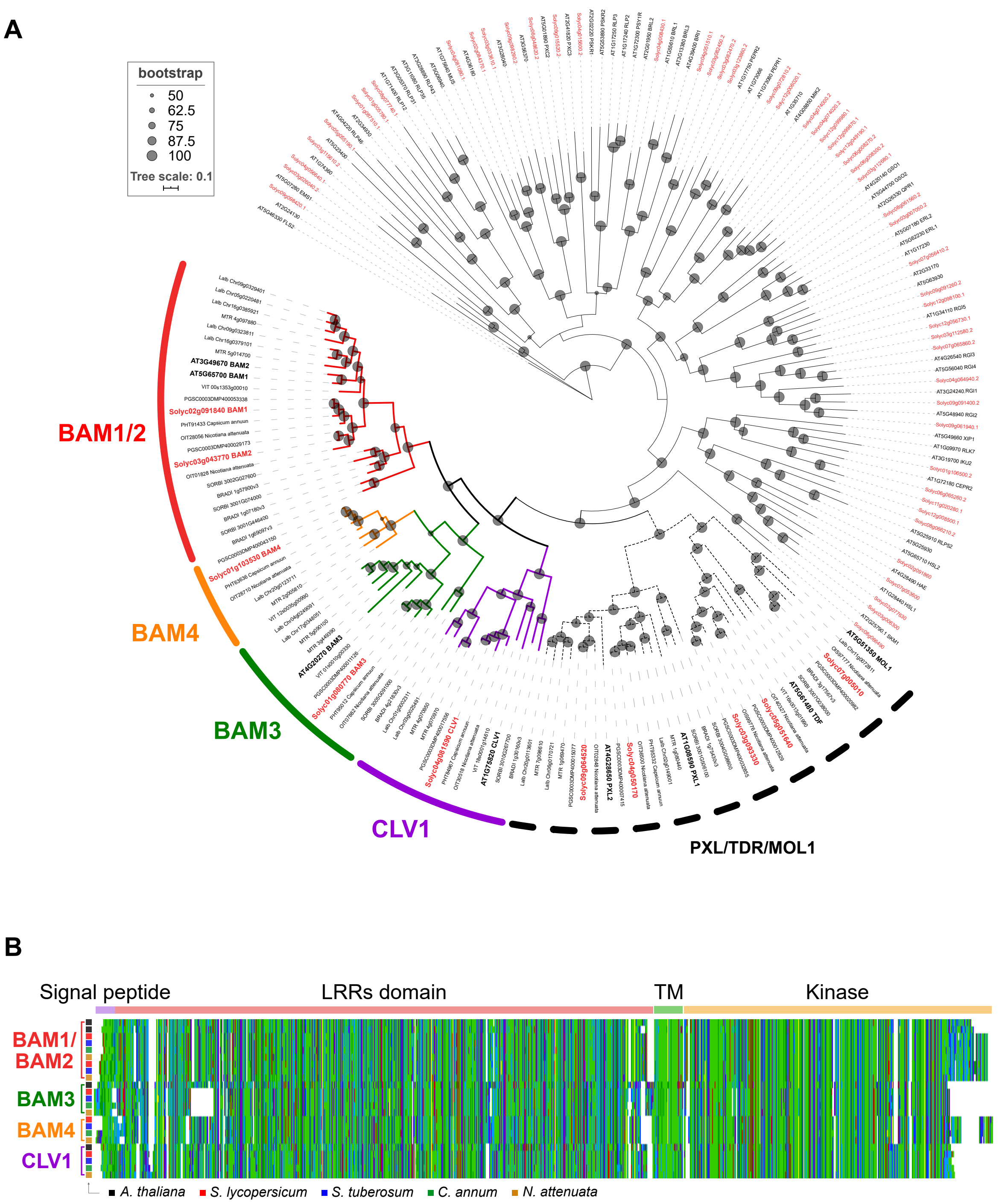

Supplemental Figure 2. Loss of *BAM4* in Arabidopsis. A. Phylogenetic tree of CLE peptide receptor extracellular domains from tomato, Arabidopsis, potato, *Capsicum annuum*, *Nicotiana attenuata*, *Lupinus albus*, *Medicago truncatula, Vitis vinifera*, *Sorghum bicolor* and *Brachypodium distachyon*. Sequences from Arabidopsis and tomato are in bold black and red, respectively. Nodes supported by bootstrap values superior to 50 are indicated by dots of size proportional to the bootstrap values. **B.** Multi-sequence alignment of BAM1/2, BAM3, BAM4 and CLV1 extracellular domains, between Arabidopsis, tomato and three additional representatives of the Solanaceae family.

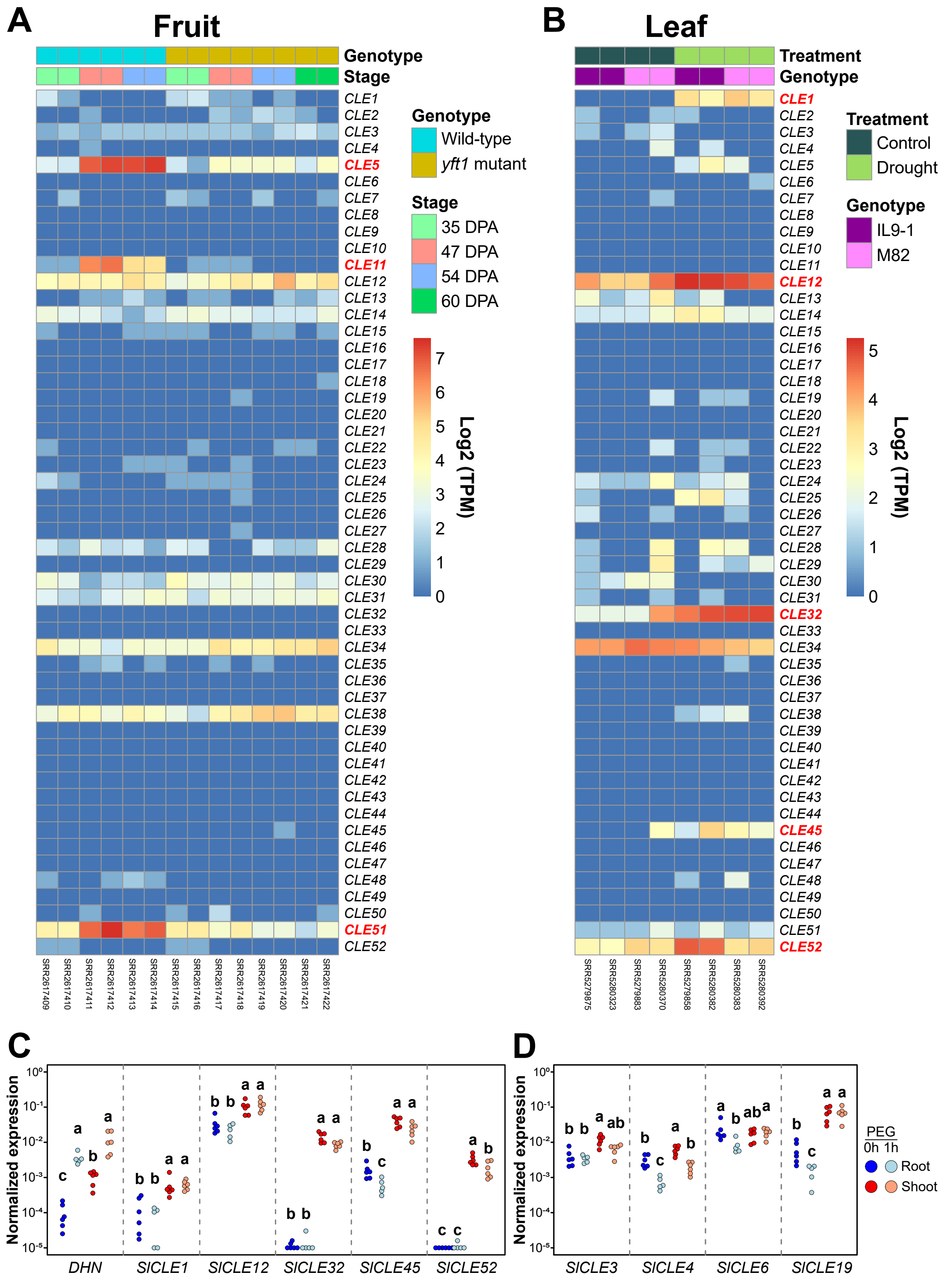

Supplemental Figure 3. Expression of tomato *CLE* genes in the developing fruit and following drought stress. A. Heatmaps of log (TPM) of *SlCLE* genes in the developing fruit at 35, 47, 54, and 60 days post antherisation (DPA), in wilt-type and *yellow fruit tomato1* mutant. Genes in red indicate *SlCLE* which are upregulated during fruit maturation in a *yft1* dependent fashion. **B.** Heatmaps of log (TPM) of *SlCLE* genes in plant leaves (M82 or a drought tolerant introgression line IL9-1) after a 10 days drought stress in pots. Genes in red indicate *SlCLE* which are upregulated by the drought treatment. **C.** Expression analysis by qPCR of selected *SlCLE* genes on root and shoot samples from 3 weeks old plants grown in

hydroponic after 1 hour 15% PEG6000 treatment. **D.** Expression analysis of the orthologs of *AtCLE25* in response to osmotic stress induced by the PEG treatment.

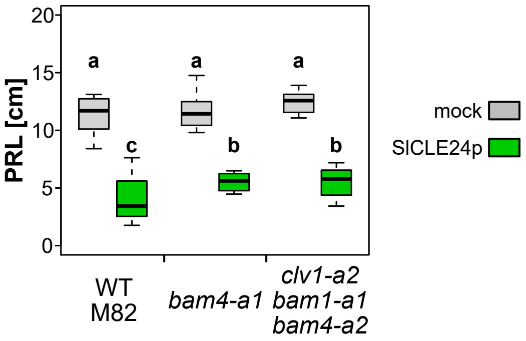

Supplemental Figure 4. Effect of SlCLE24 peptide on the primary root length (PRL) in wild type (M82) and *clv1bam1bam4* mutant.

Supplemental Table 1. Primers used in this study.

Table1. qPCR primers.

Number	Gene	Sequence	Reference
F01	*SlActin*	GGTCCTCTTCCAGCCATCC	Zhang et al., BMC 2014
F02		CCACTGAGCACAATGTTACCG	
H11	*SlDHN*	CACCATGAGGGGCAACAGCA	Kissoudis et al., Front. Plant Sci. 2017
H12		TCACCTTCATGTTGTCCAGGCATC	
E52	*SlCLE1*	TGGTGTCTTTAAGAACTTTTGCTG	Zhang et al., BMC 2014
E53		CTCTTTATCTGGAAAATCCCCTT	
E56	*SlCLE3*	CTGCTGAGATTTTAGTAAAGCCTG	Zhang et al., BMC 2014
E57		GAATGCCTTTCTGTTTCTATTATCC	
F58	*SlCLE4*	GGGAAGGGAACTAGGGCTGCCA	This study
F59		TGGCATTTGGTCCAGTAGGCACT	
E60	*SlCLE5*	AACCTCCCACTTCATTACTTCTTC	Zhang et al., BMC 2014
E61		ATGATCTGCAGCACCAGCAT	
E62	*SlCLE6*	TGGAGGTGTTACAACAAAAATGA	Zhang et al., BMC 2014
E63		GAACATGATGAGCACCACTTGA	
E74	*SlCLE12*	TGATGGATATTGATCTCTTGTGGA	Zhang et al., BMC 2014
E75		ATGAATGGTTGGGAACTGGAT	
E76	*SlCLE13*	CAATATGCAAGTCCATCACAAAC	Zhang et al., BMC 2014
E77		GCCTCCCCATAATATTTTCGA	
J08	*SlCLE19*	CCTAATGGCCCAGACCCTAT	This study
J09		GGCTTGCCAAATTCTCCTTT	
F62	*SlCLE21*	TCGTGGAGTCGAGAACATGAGGA	This study
F63		GGATCTGGTCCTCCAGGTGCAA	
F66	*SlCLE24*	AAGGCTGCTGTCGTGCAAGACC	This study
F67		CCCTGCACACTTGATTTGGACTCGC	
F78	*SlCLE32*	ACCTCTCCAAGAGTTCATGTCATCCA	This study
F79		AATATGACACTCTCTTTTCGTGGCGA	
G09	*SlCLE40*	TGTCCTCCCTCCGAAAGTCGTCC	This study
G10		TATCGTTTCCCCTCTCCTCCGCG	
G15	*SlCLE45*	TGAAAACCCCATGAGCCATGACT	This study
G16		TCCTCTTGAGAAGAAGCTTCCTTTGT	
G25	*SlCLE52*	ACCACGACCACCACTACTGTCA	This study
G26		GCAGCCACCCCATATTGCCCTC	

Supplemental Table 2. Resources and parameters used in this study.

RESOURCE	SOURCE	IDENTIFIER
Software and algorithms		
splitter -size 9000000 -overlap 1000<br>seqretsplit (defaults)<br>degapseq (defaults)	Rice et al., 2000<br>doi: 10.1016/s0168-9525(00)02024-2	https://emboss.sourceforge.net
clustalo --outfmt=fa	Sievers et al., 2011<br>doi : 10.1038/msb.2011.75	http://www.clustal.org/omega/
hmmbuild (defaults)<br>hmmconvert -2<br>hmmsearch -A --max --incT 4.0<br>hmmalign (defaults)	Eddy, 2011<br>doi: 10.1371/journal.pcbi.1002195	http://hmmer.org
genewise -both -pep -gff -hmmer	Birney et al., 2004<br>doi: 10.1101/gr.1865504	https://www.ebi.ac.uk/~birney/wise2/
bedtools multicov -bams SRR*.bam -bed<br>CLEgenepos.bed > CLEcov.tab.txt	Quinlan & Hall, 2010<br>doi: 10.1093/bioinformatics/btq033	https://bedtools.readthedocs.io/en/latest/
bwa mem -t 24 -M	Li & Durbin, 2010<br>doi: 10.1093/bioinformatics/btp698	https://bio-bwa.sourceforge.net
samtools view -b -@ 24 -t<br>samtools sort -@ 24 -T<br>samtools index	Li et al., 2009<br>doi: 10.1093/bioinformatics/btp352	https://www.htslib.org
faToTwoBit (defaults)<br>blat -t=dnax -q=prot -minIdentity=98 -fine	Kent, 2002<br>doi:10.1101/gr.229202	https://hgwdev.gi.ucsc.edu/~kent/src/
R version 4.2.1 (2022-06-23)<br>Platform: x86_64-apple-darwin17.0 (64-bit)<br>Running under: macOS Catalina 10.15.7<br><br>Matrix products: default<br>BLAS:/System/Library/Frameworks/Accelerate.framework/Versions/A/Frameworks/vecLib.framework/Versions/A/libBLAS.dylib<br>LAPACK:/Library/Frameworks/R.framework/Versions/4.2/Resources/lib/libRlapack.dylib<br><br>locale: fr_CH.UTF-8/fr_CH.UTF-8/fr_CH.UTF-8/C/fr_CH.UTF-8/fr_CH.UTF-8<br><br>attached base packages:<br>stats / graphics / grDevices / utils / datasets / methods / base<br><br>other attached packages:<br>pheatmap_1.0.12 rlang_1.0.6<br><br>loaded via a namespace (and not attached):<br>Rcpp_1.0.9 / plyr_1.8.7 / grid_4.2.1 / R6_2.5.1 / lifecycle_1.0.3 / gtable_0.3.1 / magrittr_2.0.3 / XLConnect_1.0.5 / scales_1.2.1 / stringi_1.7.8 / cli_3.4.1 / reshape2_1.4.4 / farver_2.1.1 / rstudioapi_0.14 / RColorBrewer_1.13 / tools_4.2.1 / stringr_1.4.1 / munsell_0.5.0 / compiler_4.2.1 / colorspace_2.0-3 / BiocManager_1.30.18 / rJava_1.0-6		https://cran.r-project.org/
MegaX_10.1.8 (defaults)	Kumar et al., 2018	https://www.megasoftware.net/
IQ-TREE (defaults)	Trifinopoulos et al., 2016	http://iqtree.cibiv.univie.ac.at/

Supplemental table 3. The extended information about *SlCLE* genes. Can be downloaded as an Excel file.
